# Supplementary material for: Dietary fat promotes antibiotic-induced Clostridioides difficile mortality in mice
Source: NPJ Biofilms Microbiomes. 2022 Apr 1;8:15. doi: 10.1038/s41522-022-00276-1 (PMC8975876; doi:10.1038/s41522-022-00276-1)
Supplement: Supplementary file 1 — Supplementary Materials [file 41522_2022_276_MOESM1_ESM.docx]

**Supplementary Materials** for Hazleton K.Z., et al. *Dietary fat promotes antibiotic-induced Clostridioides difficile mortality in mice*


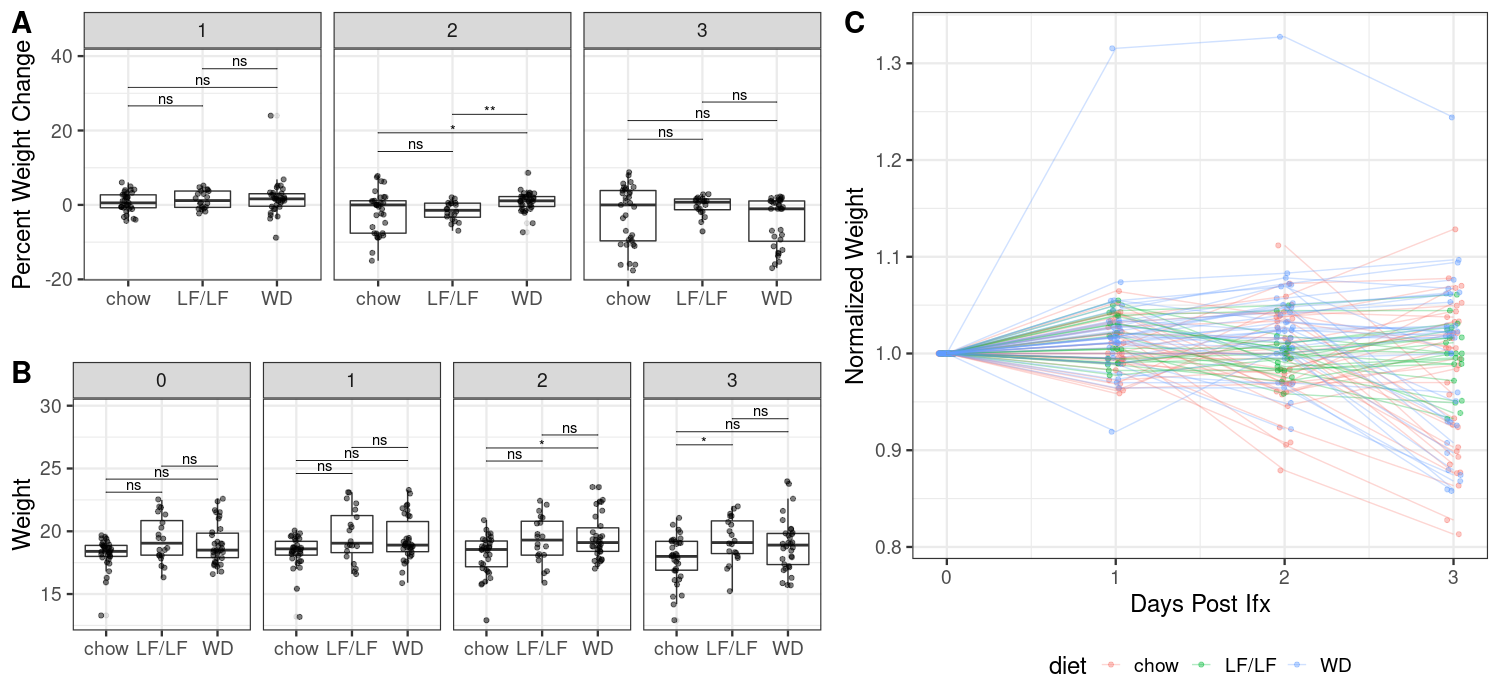


**Supplementary Figure 1:** Weight of mice before and after infection with *C. difficile* in Cohort 1. (A) Percent weight change of mice after 1,2, and 3 days of infection across diets (baseline was day of infection); (B) weight in grams of mice across diets pre and 1,2, and 3 days post infection and (C) scatter plot of fractional weight change of each mouse, colored by diet. p-values were determined using a Kruskal-Wallis with Dunn’s post hoc test. Median and IQR indicated. (* : p < 0.05, ** : p < 0.01).

**
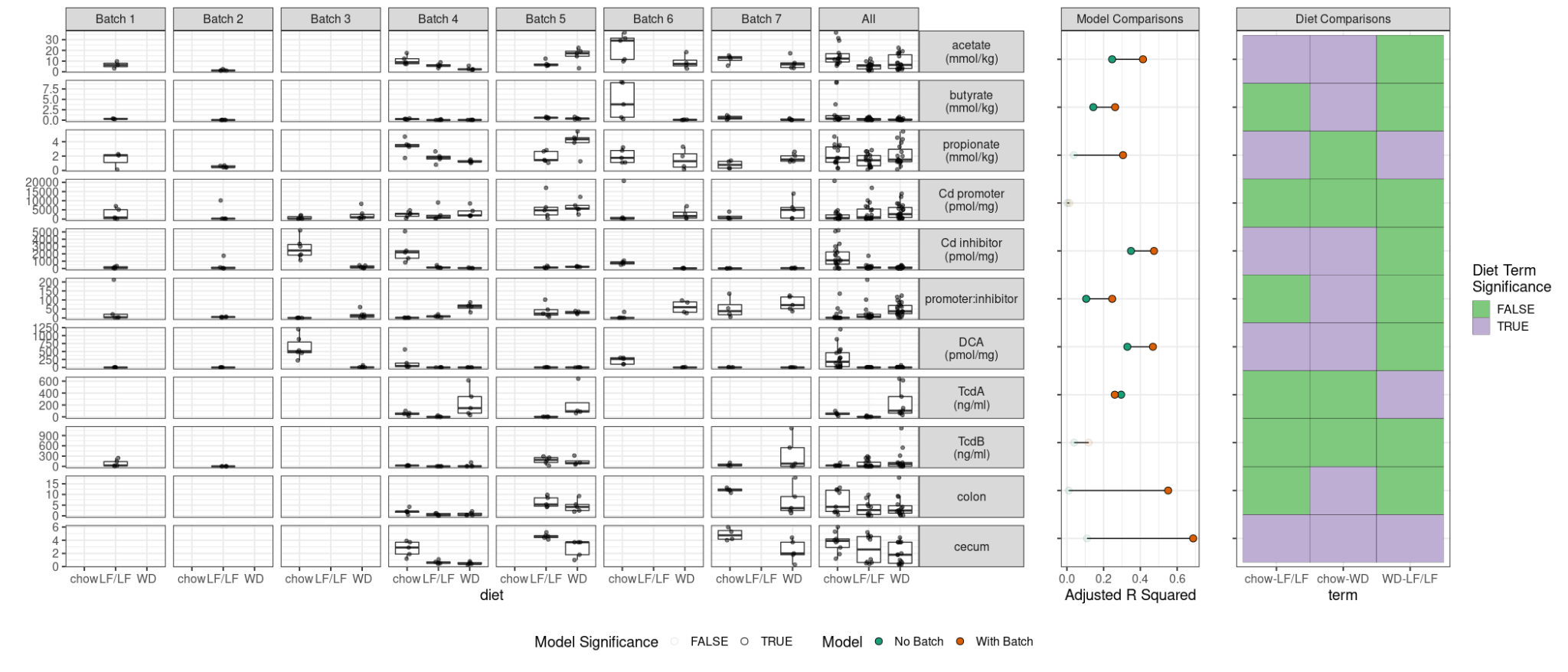
**

**Supplementary Figure 2:** Analyte concentrations and inflammatory scores across different batches. The middle panel compares the adjusted R squared of linear models without considering batch (readout ~ diet) to linear models while considering batch (readout ~ diet + batch). Models are opaque if the corrected p values < 0.05 and translucent if not. The right panel shows significant differences between diet groups for models that consider batches.


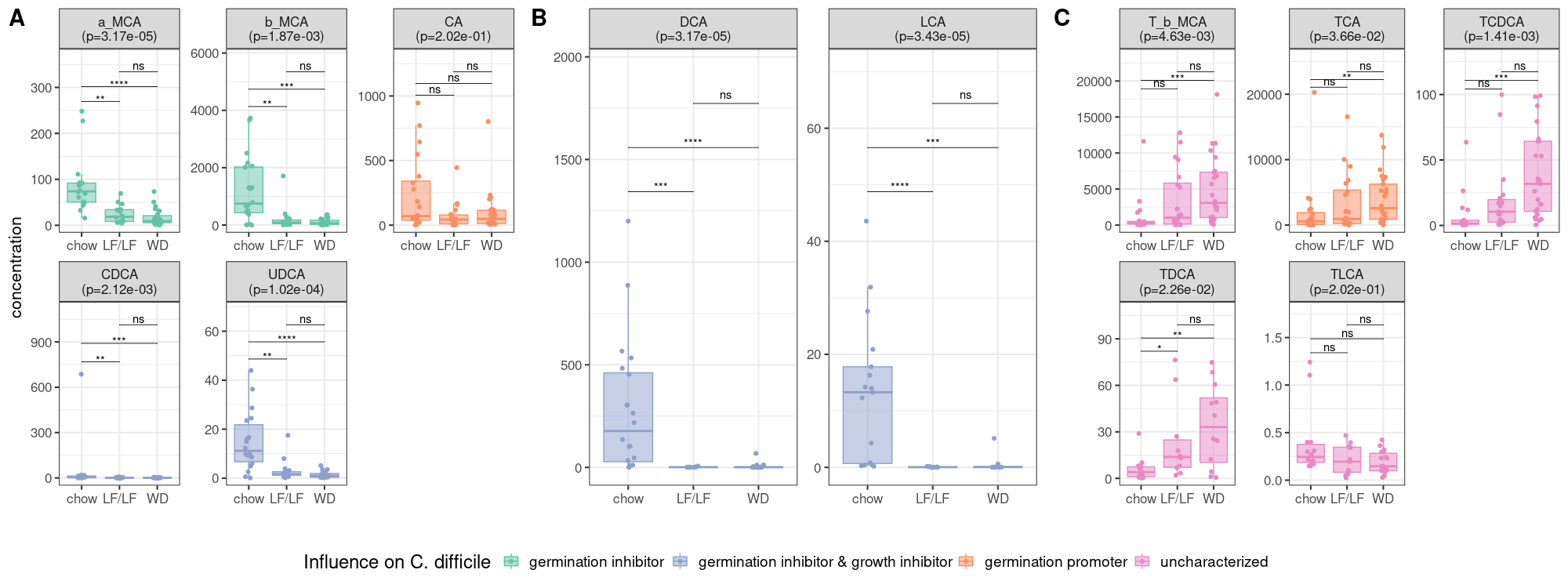


**Supplementary Figure 3.** Cecal bile acid concentrations (pmol/kg) by diet. Unconjugated primary bile acids (A), secondary bile acid (B) and taurine-conjugated bile acids (C). a_MCA (alpha muricholic acid); b_MCA (beta muricholic acid); CA (cholic acid); CDCA (chenodeoxycholic acid); UDCA (ursodeoxycholic acid); DCA (deoxycholic acid); LCA (lithocholic acid); T_b_MCA (tauro-beta muricholic acid); TCA (taurocholic acid); TCA_3_SO4 (taurocholic acid 3-sulfate); TCDCA (taurochenodeoxycholic acid); TDCA (taurodeoxycholic acid); TLCA (taurolithocholic acid). P-values for the Kruskal-Wallis test with Dunn’s post hoc are noted. Median and IQR are indicated. Plots are colored based on the previously described influence of each bile acid on *C. difficile*.


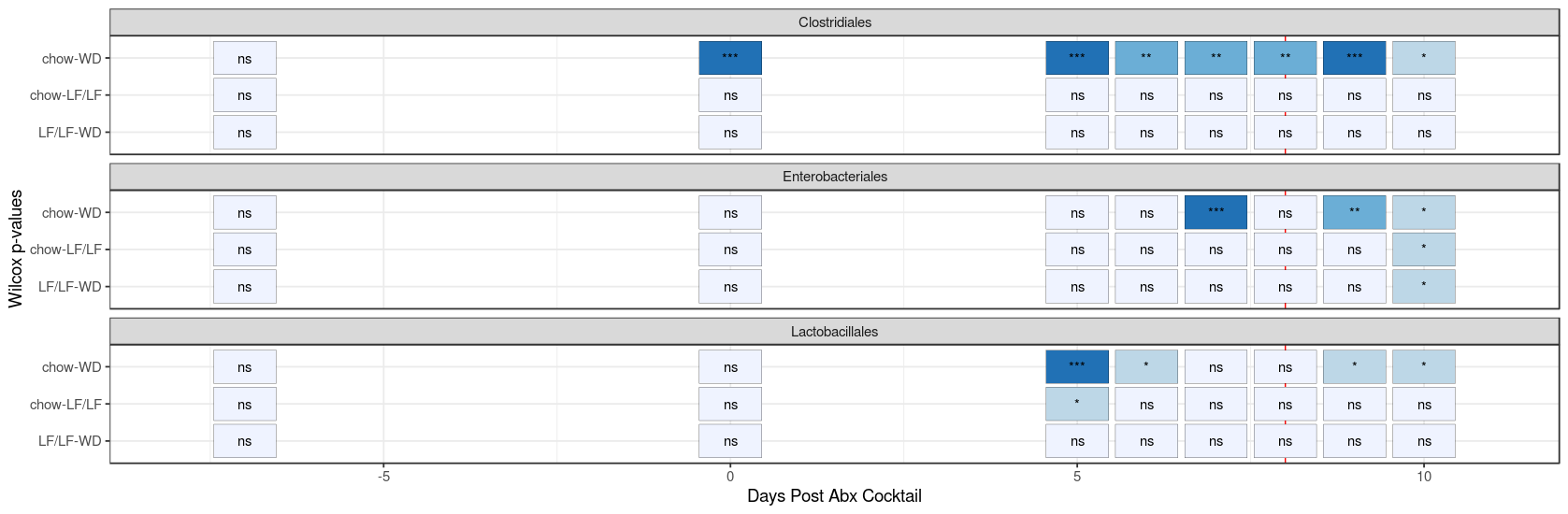


**Supplementary Figure 4:** Statistical summary of relative abundances of key taxa from Figure 7a. Significant differences between groups are noted as calculated with the Wilcoxon rank-sum test. ***: p<0.001. **: p<0.01, *: p<0.05, ns= non significant.

**Supplementary Table 1**: Composition of low-fat/low-fiber/high-sucrose diet

|  | ***Low-fat/Low-fiber/High-Sucrose*** |
| --- | --- |
| Fat (% kcal)  *(% SFA)*  *(% MUFA)*  *(% PUFA)* | 17.0  *(19.5)*  *(41.7)*  *(38.8)* |
| Carbohydrates (% kcal)  *(Sucrose)* | 64.5  *(26.7)* |
| Protein (%kcal) | 18.6 |
| Fiber (g/kg) | 50 (cellulose) |

**Supplementary Table 2:** Description of sample sizes across different assays in cohorts 1 and 2.

|  |  | Batches | Chow | LF/LF | WD |
| --- | --- | --- | --- | --- | --- |
| Cohort 1 *  (stool collection) |  | Total  Batch1  Batch2  Batch3  Batch4 | 20  10  10  0  0 | 20  0  10  5  5 | 20  10  0  5  5 |
|  | 16S rRNA | Batches 1,2,3 | 13 | 5 | 13 |
| Cohort 2 **  (tissue collection) |  | Total  Batch1  Batch2  Batch3  Batch4  Batch5  Batch6  Batch7 | 20  0  0  6  5  0  5  4 | 20  5  5  0  5  5  0  0 | 26  0  0  6  5  5  5  5 |
|  | histology | Batches 4,5,7 | 9 | 10 | 14 |
|  | TcdA | 4,5 | 5 | 10 | 9 |
|  | TcdB | 1,2,4,5,7 | 8 | 20 | 15 |
|  | Bile acids | All batches | 20 | 20 | 25 |
|  | SCFAs | 1,2,4-7 | 14 | 18 | 19 |

*Cohort 1 (longitudinal mortality analysis with serial fecal collection and microbiome sequencing) was conducted in 4 different batches of mice with per batch counts across diets indicated.

**Cohort 2 (tissue collection – intestines for histopathology and cecal aspirates for bile acids, SCFAs, and toxins) was conducted in 7 different batches of mice with per batch counts across diets indicated. 16S rRNA, histology, toxins TcdA and TcdB, bile acids and SCFAs were all measured for particular sets of batches as indicated.

**Supplementary Table 3**

Complete results of linear modeling statistical analyses performed. **[Table in separate Excel spreadsheet file]**

**Supplementary Table 4**

| Ingredient (g/KG) | WD | LF/LF |
| --- | --- | --- |
| Anhydrous Milkfat | 36.3 | 50 |
| Beef Tallow | 24.8 | 0 |
| Casein | 190 | 0 |
| Cellulose | 30 | 35 |
| Cholesterol | 0.4 | 0 |
| Choline Bitartrate | 2.1 | 0.01 |
| Corn Oil | 16.5 | 6.5 |
| Corn Starch | 230 | 392.2 |
| L-Cystine | 2.85 | 3 |
| Lard | 28 | 0 |
| Maltodextrin | 70 | 100 |
| Mineral Mix, nTWD (110422) | 35 | 15 |
| Olive Oil | 28 | 16.5 |
| Sodium Chloride | 4 | 0 |
| Soybean Oil | 31.4 | 23 |
| Sucrose | 255.6 | 24 |
| TBHQ, antioxidant | 0.028 | 0.014 |
| Thiamin (81%) | 0.015 | 0.002 |
| Vitamin K1, phylloquinone | 0.003 | 0.014 |
| Vitamin Mix* | 15 | 2.75 |

* WD used the Vitamin Mix nTWD (110423) and LF/LF diet used AIN-93-VX (94047)
